# Non-Surgical Management of Malignant Bowel Obstruction in Advanced Ovarian Cancer patients: A Systematic Review and Meta-Analysis

**DOI:** 10.1177/10499091211043079

**Published:** 2021-09-07

**Authors:** Praveena Idaikkadar, Athina Georgiou, Simon Skene, Agnieszka Michael

**Affiliations:** 1 Faculty of Health and Medical Sciences, University of Surrey, Guildford, United Kingdom

**Keywords:** ovarian cancer, terminal care, intestinal obstruction, prognosis, quality of life, meta-analysis

## Abstract

**Background:**

Malignant bowel obstruction is a common cause of morbidity and mortality in patients with advanced ovarian cancer. Many patients aren’t suitable for, or decline, surgical decompression. The outcomes for this frail group of patients are not well characterized.

**Aim:**

To evaluate survival outcomes of ovarian cancer patients who undergo non-surgical management of malignant bowel obstruction.

**Design:**

Systematic review and meta-analysis.

**Data Sources:**

Online literature search of Pubmed, Embase and Medline libraries up until December 2020. Searching abstracts of scientific meetings, reference lists of included studies and contacting experts in the field.

**Selection Criteria:**

Studies that investigated non-surgical management of confirmed bowel obstruction in advanced ovarian cancer patients were included. All levels of evidence including RCTs, cohort studies and case-series if they included greater than 5 patients.

**Data Collection and Analysis:**

The studies were independently chosen by two reviewers who extracted and analyzed the data separately through OpenMeta Analyst software. Study quality was assessed using the JADAD score and the Newcastle Ottawa Score.

**Results:**

24 studies met the eligibility criteria for the systematic review and 9 for the meta-analysis. Median survival of patients managed non-surgically for bowel obstruction was 44 days (95% CI 38-49 days, I ^2^ = 0%, P = 0.128).

**Conclusion:**

The quality of studies was relatively low, however the evidence shows that non-surgical management of bowel obstruction results in a short life expectancy but with controlled symptoms. Where quality of life is the main concern, this may be a feasible and effective strategy.

## Introduction

Ovarian cancer is the most lethal gynecological malignancy and the sixth most common cancer among women globally.^1^ Most patients present at an advanced stage of disease when the cancer has spread widely and is no longer curable. Malignant bowel obstruction (MBO) can be defined as a mechanical or functional obstruction of the small or large intestine that prevents physiological transit and digestion, caused by cancer within the abdomen, either primary or metastatic.^2^ The incidence of MBO in patients with cancer of any primary etiology is approximately 2%,^3^ however it is more prevalent in cancers of the gastrointestinal (GI) or gynecological tract and in patients with ovarian cancer the incidence ranges from 5% to 51%.^4^ This can be attributed to the mode of spread of ovarian cancer, which is typically direct invasion of adjacent organs and peritoneal carcinomatosis through the trans-coelomic route, rather than hematogenous or lymphatic spread. MBO may originate in the small bowel (61%), large bowel (33%) or in both simultaneously (20%). It can be complete or partial, and may involve one or multiple levels.^2^ The morbidity and mortality associated with MBO is significant and is often associated with advanced disease that may have become chemotherapy resistant and can become a recurring feature of the disease or signal the end of life. It presents a very distressing scenario for patients, their families and clinicians.

Management of MBO in advanced ovarian cancer can be divided into 2 pathways: surgical management and medical management, both of which include palliative procedures to improve symptoms and quality of life and in some cases, length of life. Surgical management can involve either direct resection of tumor tissue/bowel or bypass surgery to allow transit of material around the obstruction, in some cases a stoma may be formed, either temporary or more often, permanent. Non-surgical or medical management of bowel obstruction includes endoscopic procedures such as stents, percutaneous or radiologically inserted gastrostomy, nasogastric or nasojejunal tubes for decompression and bowel rest. Parenteral feeding including IV fluids and total parenteral nutrition (TPN) is important to maintain nutrition. Medication for symptom control is integral to the overall management of MBO regardless of whether surgery is performed or not. It includes chemotherapy, steroids and supportive medication such as antisecretory drugs (including octreotide and scopolamine), analgesia, anti-emetics and importantly, a medication review to stop all pro-kinetic drugs.^5^

The rationale in choosing between surgical or medical management strategies is not well defined and should be personalized to the individual patient and delivered by a multi-disciplinary team including surgeons, oncologists, palliative care physicians, dietitians and other allied health partners. Key factors in determining whether a patient should be offered surgery have been investigated but to date no single scoring system has been effectively validated.^6,7^ Factors which should be considered can be subdivided into patient factors and disease factors. Patient factors include the patients performance status, overall prognosis including availability and suitability of further systemic anti-cancer therapy, other co-morbidities and patient choice.^4^ Disease factors that can affect operability include whether it is a single site obstruction or multi-level, partial or complete obstruction and other non-malignant adhesions within the abdominal cavity. However, peri-operative morbidity (9%-90%) and mortality (9%-40%)^4,8^ can make surgery a risky choice and the practice of offering surgery varies widely between different cancer centers.^9^ There is increasing evidence that non-surgical management can significantly improve symptoms and quality of life.

## Objectives

To evaluate the outcomes of patients with advanced ovarian cancer who undergo non-surgical management of malignant bowel obstruction and conduct a meta-analysis to estimate median survival from diagnosis of bowel obstruction.

## Methods

### Inclusion Criteria for Study Entry

- Types of studies: We searched for original research papers of all levels of evidence including randomized controlled trials (RCTs), retrospective and prospective cohort studies, case control studies and case series (as long as they included greater than or equal to 5 patients). Review articles were not included. The number and quality of studies was low and so, less stringent inclusion criteria were chosen to be more inclusive and concentrate on “best evidence available.” Only studies written in English and published after 1980 were included. Conference abstracts were not included as the level of data analysis was found to be inadequate.
- Types of participants: Adult patients with a clinical diagnosis of advanced or recurrent ovarian cancer (or primary peritoneal cancer or fallopian tube cancer) who had radiologically confirmed or classical symptoms of bowel obstruction, and who did not undergo an operation. Studies that included patients with non-ovarian cancer (such as other gynecological or bowel cancer) were not included unless the data for the ovarian cohort alone could be abstracted.
- Types of intervention: any non-surgical procedure performed to improve symptoms such as a stent or tube placement; medications such as steroids, chemotherapy, analgesia, anti-emetics, anti-secretory agents, e.g. octreotide.
- Types of outcome measures: The primary outcome for the meta-analysis was median survival from the time of diagnosis of bowel obstruction. Studies which did not report on this (for example reported on quality of life outcomes or symptom control) were limited to the systematic review.

### Search Methods for Identification of Studies

- Electronic Searches: 3 online databases (Pubmed, Embase and Medline) were searched up until December 2020. The following search string was used, combining key search terms with Boolean Operators—(Ovary cancer) OR (Ovary Carcinoma) OR (Ovarian Cancer) OR (Ovarian Neoplasm) AND (Intestine Obstruction) OR (Colon Obstruction) OR (Small Intestine Obstruction) OR (Intestine Occlusion) OR (Bowel Obstruction) AND (Non-surgical management) OR (Medical Management) OR (Conservative Management) OR (palliative management).
- Relevant articles were then found on Pubmed and further studies were identified through the “similar articles” feature.
- Hand searching: based on the results of the above searches, key journals were specifically searched for studies of interest. Citation lists of retrieved articles and noteworthy reviews were also assessed and used to find further relevant articles.

## Data Collection and Analysis

### Selection of Studies

Two authors independently conducted the literature search and used the inclusion criteria to assess the suitability of each study. The studies from the online searches were collated via NICE’s Healthcare Databases Advanced Search, duplicates removed and a “title and abstract” screening used to assess relevance at which point the full text was obtained. For any studies where relevance could not be clearly differentiated from the abstract alone, the full text was obtained to clarify. Differences in opinion between the two authors could be resolved by a third author but this was not necessary.

### Data Extraction

Data was independently extracted by the two authors using a specifically designed data collection form and the following information retrieved:

– First author
– Year of publication
– Country of study
– Inclusion and exclusion criteria
– Study Type
– Type of intervention
– Sample size (number of patients)
– Average age
– FIGO Stage of cancer
– Ovarian cancer histology and grade
– ECOG Performance status
– Median survival from diagnosis of bowel obstruction
– Standard error or 95% confidence interval

### Statistical Analysis

Meta-analysis was carried out using the OpenMeta Analyst software^10^ to calculate the pooled median survival in days from the diagnosis of bowel obstruction with 95% confidence interval. The analysis was undertaken using a fixed effects model, with heterogeneity assessed by reference to I^2^. In the event of significant heterogeneity meta-analysis would be repeated using a random effects model to account for variability between studies.

## Results

### Results of the Search

**Figure 1.**
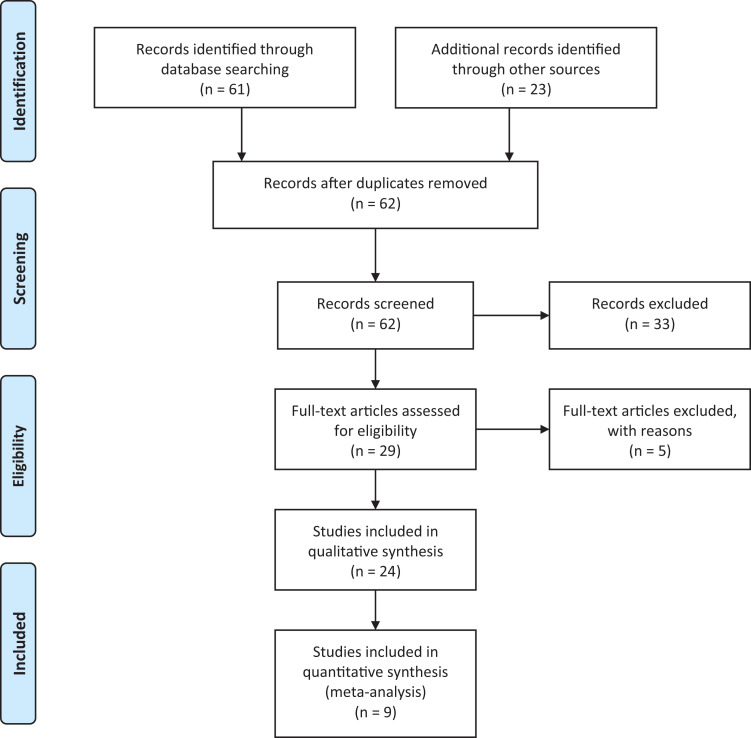
PRISMA flow diagram to show the process of the literature search and relevant results.

The electronic search strategy identified 61 articles, when duplicates were removed there were 39 articles of potential interest. This was supplemented with the other search strategies mentioned above, primarily citation screening of relevant articles, which added a further 23 articles. Title and abstract screening left 29 possibly relevant articles, five articles were excluded on full text screening: three were conference abstracts with no associated literature publication, one was a mixed population of patients including pancreatic cancer and it was impossible to differentiate the data only for the ovarian cohort, and one paper had a cohort of patients where some also had surgery. This left 24 relevant articles.^11-34^ Only 9 of the 24 articles had sufficient statistical data (either standard error or 95% confidence intervals for the median OS, or the raw data itself) to facilitate inclusion in the meta-analysis. These results are presented in Figure 1—PRISMA flow diagram.^35^

### Study Characteristics

Table 1 summarizes the characteristics of the 24 included articles. In terms of study design, one was an RCT, 3 were prospective case series, 8 were retrospective case series and 12 were retrospective cohort studies. Most were conducted in Europe (9) and the USA (12), two studies were from Canada and one from China. The studies tended to be a little older, ranging from 1986 to 2017. In 13 studies, medical intervention was being compared to surgical intervention but only the data regarding non-surgical management was included in the review and meta-analysis. Of medical management approaches, three papers assessed the efficacy of chemotherapy, two focused on stents, eight on PEG tubes, one paper on TPN, 3 papers on Octreotide and eight papers looked at a variety of combination supportive medical managements. In nine papers the study characteristics, patient population and outcomes reported were similar enough to be meaningfully combined into a meta-analysis of the primary outcome—median survival from time of obstruction. The total number of patients analyzed was 2236 for the systematic review and 364 for the meta-analysis.

**Table 1.** Characteristics of the 24 Studies Included in the Systematic Review.

First author	Year	Country	Study design	Type of intervention	Number of patients
Dean	2017	UK	Retrospective cohort	Chemotherapy	129
Milek	2017	Poland	Retrospective case series	Stent	13
Jolicoeur	2003	Canada	Retrospective case series	PEG	24
Abu-Rustum	1996	USA	Retrospective case series	Chemo + PEG	21
Brard	2005	USA	Retrospective cohort	TPN	55
Gadducci	1998	Italy	Retrospective cohort	Medical	34
Zoetmulder	1994	Netherlands	Retrospective cohort	Medical	58
Mangili	2005	Italy	Retrospective cohort	Octreotide	47
Bais	1995	Netherlands	Retrospective cohort	Medical	31
Bryan	2006	USA	Retrospective cohort	Chemotherapy	39
Mangili	1996	Italy	Retrospective case series	Octreotide	13
Peng	2015	China	RCT	Octreotide vs Scopolamine	96
Matulonis	2005	USA	Prospective case series	Long acting Octreotide	13
Rath	2013	USA	Retrospective case series	PEG	53
Chi	2009	USA	Prospective case series	PEG or Stent	26
Pothuri	2005	USA	Retrospective case series	PEG	94
Mooney	2012	USA	Retrospective cohort	Medical	1145
Suidan	2017	USA	Retrospective cohort	PEG	154
Malone	1986	USA	Prospective case series	PEG	10
Larson	1988	USA	Retrospective case series	Medical	33
Beattie	1989	UK	Retrospective cohort	Medical	43
Fernandes	1987	Canada	Retrospective cohort	Medical	62
Redman	1988	UK	Retrospective cohort	Medical	38
De Eulis	2015	USA	Retrospective case series	PEG	5

### Patient Characteristics

The median age of patients included was 57. In six of the studies the median age could not be calculated but it is worth noting that in three of these studies the American SEER database was used which only holds details of patients aged over 65 years which is higher than the average age of patients in the other included studies. The vast majority of patients had FIGO stage III or IV disease and serous histology (where specified in only 10 studies). There was very little reporting of grade of tumor (10 studies) and patient performance status (4 studies). See Tables 2 and 3.

**Table 2.** Patient Characteristics—Age, FIGO Stage and Histology of Ovarian Tumor.

First author	Average age	Initial FIGO Stage (5)					Histology					
		I	II	III	IV	Unknown	Serous	Endometrioid	Clear cell	Mucinous	Undifferentiated	Other
Dean	61	6		67	18	9	44	2	7	2	19	43
Milek												
Jolicoeur												
Abu-Rustum	55		5	76	14	5						
Brard	56			100								
Gadducci	60	3	6	58	33		63	4	3	12	16	1
Zoetmulder	55	5	5	71	7	10						
Mangili	60		4	85	11							
Bais	54		13	67	19		45	13	3	16	23	
Bryan	55	5	2	85	8		65	5	8	3	8	11
Mangili	57			100			85			8		8
Peng	54			19	81							
Matulonis	58											
Rath	60	8	4	66	19	4						
Chi	54											
Pothuri	56	1	2	70	27							
Mooney							58	6		5		30
Suidan		0	7	53	34		75					25
Malone				100			90	10				
Larson	59	0	15	67	9	9						
Beattie		5	5	80	15							
Fernandes	61	10		61	29							
Redman	57	3	24	62	11							
De Eulis	61	0	0	60	40							

**Table 3.** Patient Characteristics—Grade of Tumor and ECOG Performance Status.

First Author	Grade				ECOG PS			
	1	2	3	Unknown	0-1	2	3+	?
Dean				100	24	26	19	31
Milek								
Jolicoeur								
Abu-Rustum								
Brard	2	51	47		2	85	13	
Gadducci								
Zoetmulder								
Mangili					70	30		
Bais	6	6	77	10				
Bryan	3	18	59	20				
Mangili								
Peng								
Matulonis								
Rath	6	4	87	4				
Chi					85	8	8	
Pothuri	2	25	68	5				
Mooney								
Suidan	16		57	27				
Malone		100						
Larson								
Beattie								
Fernandes	26	23	35	16				
Redman								
De Eulis	0	60	40					

### Outcomes Reported

For the 9 studies included in the meta-analysis the median overall survival from time of diagnosis of bowel obstruction or first intervention, was recorded. A variety of other outcome measures were also used including attempts to measure quality of life, such as number of days to symptom relief and proportion of patients who stopped vomiting / were able to open their bowels normally / what level of oral intake they could tolerate. This is an equally important outcome measure as this group of patients have incurable disease and therefore quality of life, not just length of life, is a crucial factor. A few studies also reported on the proportion of patients who were able to improve to the point of discharge or were able to restart more chemotherapy, which again is a very clinically relevant outcome.

### Methodological Assessment of Study Quality

The risk of bias in the studies was assessed using the JADAD score for the only RCT (Peng et al)^21^ which scored 3/5 on the JADAD score. The Newcastle Ottawa Scale (NOS) was used for the observational studies and the results are presented in Table 4.

**Table 4.** Results of the Newcastle Ottawa Scale (NOS) for Observational studies. For Cohort Studies, 8 Domains Are Represented With a Total Possible Score of 9. For Case Series 6 Domains Are Represented (The Other 3 Domains Shaded Out) With a Total Possible Score of 6.

Study	Representativeness of exposed cohort	Selection non-exposed cohort	Ascertainment of exposure	Outcome of interest	Comparability	Assessment of outcome	Length of follow up	Adequacy of follow up	Total score (6 or 9)
Dean	*		*			*	*		4/6
Milek			*			*	*	*	4/6
Jolicoeur	*		*			*	*	*	5/6
Abu Rustum	*		*			*	*	*	5/6
Brard	*	*	*		*	*	*	*	7/9
Gadducci	*	*	*	*		*	*	*	7/9
Zoetmulder	*	*	*		*	*	*	*	7/9
Mangili 2005	*	*	*			*	*	*	6/9
Bais	*	*	*			*	*	*	6/9
Bryan	*	*	*			*	*	*	6/9
Mangili 1996			*			*	*	*	4/6
Matulonis			*			*	*	*	4/6
Rath	*		*			*	*	*	5/6
Chi	*		*			*	*	*	5/6
Pothuri	*		*			*	*	*	5/6
Mooney			*			*	*	*	4/6
Suidan			*			*	*	*	4/6
Malone			*			*	*	*	4/6
Larson	*		*			*	*	*	5/6
Beattie	*		*			*	*	*	5/6
Fernandes	*	*	*		*	*	*	*	7/9
Redman	*	*	*			*	*	*	6/9
De Eulis			*			*	*	*	4/6

For studies which were presented as case series, the total possible score on the NOS was 6. All studies in this group scored 4 or 5 out of 6—indicating a “Fair” quality or “Poor” quality. The studies that ranked “Poor” all lost points due to the “representativeness of the cohort”, often this was because the patient group was selective. For example, 3 papers recruited historic patients from the SEER database which only registers patients over the age of 65.

For studies that had a “cohort” study design, the maximum possible score was 9 and all studies scored either 6 or 7 out of 9—indicating that they were of either “Good” or “Poor” quality. The difference between the “Good” and “Poor” studies was always in the “comparability” domain, with the study design of the “Poor” studies not controlling for confounding factors such as the surgical cohort of patients having a higher performance status and longer expected life expectancy than the medical cohort.

It is important to acknowledge that, although almost all studies drew upon a representative population sample, some studies dated back to the 1980s/90 s and so patients were treated according to historic standards of care, with management techniques which are outdated. For example, in many studies around 50% patients were suboptimally debulked, many had abdominal radiotherapy and many were not treated with standard doublet platinum containing chemotherapy.

### Primary Outcome

**Figure 2.**
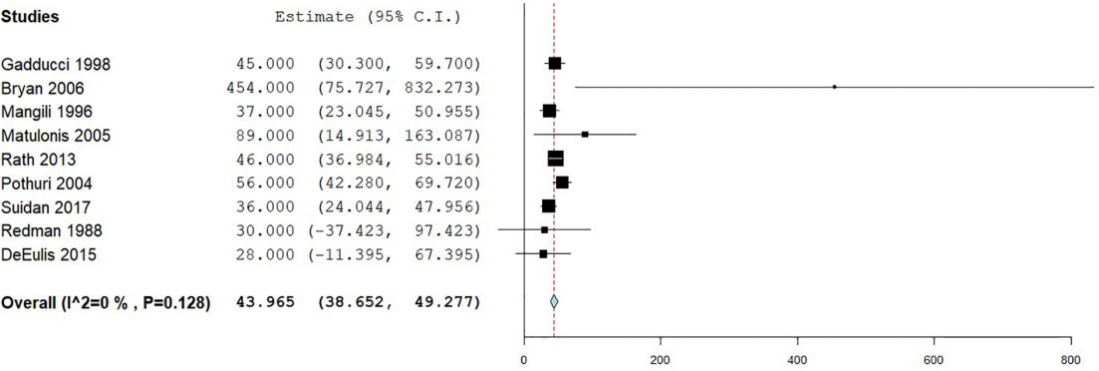
Forest plot to show median overall survival in days.

The primary outcome for the meta-analysis was median overall survival from time of diagnosis of bowel obstruction. Nine studies involving 364 patients were analyzed and the median OS was 44 days (range 28-254 days) with 95% CI 38-49 days, p = 0.128. The median survival from each paper is presented in Table 5 and a Forest Plot of the combined results in Figure 2.

**Table 5.** Median Overall Survival From Time of Bowel Obstruction, Measured in days, for the 9 Studies Included in the Meta-Analysis.

Study name	Number of patients	Median OS (Days)	95% CI	SE
Gadducci	12	45	30-60	7.5
Bryan	8	454	119-884	193
Mangili 1996	13	37	23-51	7.12
Matulonis	13	89		37.8
Rath	53	46	32-50	4.6
Pothuri	94	56	42-70	7
Suidan	154	36	15-39	6.1
Redman	12	30	15-150	34.4
DeEulis	5	28		20.1

The other studies which reported median overall survival but without enough statistical data to be included in the meta-analysis are presented in Table 6. These studies showed similar results with median OS ranging from 35 to 98 days.

**Table 6.** Median Overall Survival From Time of Bowel Obstruction, Measured in Days, for the Studies With Not Enough Supporting Data to be Included in Meta-Analysis.

Study name	Number of patients	Median OS (Days)
Dean	50	44.5
Jolicoeur	24	42
Abu-Rustum	21	84
Brard	28	72
Bais	12	37
Chi	12	78
Mooney	1145	98
Malone	10	35
Larson	14	92
Beattie	32	93

### Heterogeneity Testing

Heterogeneity testing showed there was no heterogeneity among the studies as I^2^ = 0% (p = 0.128), therefore the fixed effects model was retained.

The study reported by Bryan et al^29^ was an outlier as median OS was very high at 454 days. This was a small study with only 8 patients in the investigational arm, which considered the effect of chemotherapy. The source population was well balanced and although it is a slightly more recent study (reviewing patients treated between 1990-2003) it is not clear why survival was so long.

## Discussion

### Main Findings of the Study

The results presented in this meta-analysis show that the overall survival for patients with advanced ovarian cancer who develop malignant bowel obstruction which is managed medically, is poor at just 44 days.

The meta-analysis suggests that the choice of non-surgical management, be it chemotherapy, stent, PEG or other supportive care, makes very little difference to the overall outcome as shown by the homogeneity in the Forest Plot in Figure 2. Thus, it would seem sensible that the management plan is tailored to the needs of the individual patient. As is standard practice, all patients, especially those coming to the end of life, should be managed within a multidisciplinary setting that takes a holistic view of the patient and their needs.

Compared to survival results reported in the literature of patients managed surgically, which is often in the range of a few months,^5,7,36^ these results do show a slightly lower overall median survival. However, it is very difficult to disentangle the confounding effects of younger age and better performance status that are often present in patients offered surgery, who are also commonly less intensively pre-treated. A Cochrane review of surgical versus medical management of malignant bowel obstruction found only one paper that utilized multivariable statistical adjustment for the baseline case mix.^37^ Although there will of course be a subset of patients who benefit from a more aggressive treatment strategy, it is unclear what predictive factors can help us identify those patients and this review shows that non-surgical management can result in similarly good outcomes

### Strengths and Limitations of the Study

As with any review there will be potential biases in the review process. This has been limited by a comprehensive literature search and independent data abstraction and analysis by 2 authors. Overall, the quality of evidence found was medium to low. Almost all studies were retrospective in nature, and only one study was randomized. However, the value of cohort studies and case series is not to be under-estimated given the inclusive nature of the study design and the representative sample population that will likely closely resemble “real world data.” Patient characteristics were very similar between the included studies and this was reflected in the homogeneity of the outcomes.

Although, overall, the inclusion criteria for each individual study were broad, they were not identical between the studies. For example, most but not all studies, excluded patients who were within 30 days of their initial diagnosis, as these patients represent a very different population than those with longstanding or recurrent ovarian cancer, and in these patients bowel obstruction may be an iatrogenic complication of their initial debulking surgery.

It was unfortunate that many otherwise good quality studies could not be included in the meta-analysis due to incomplete reporting of data. This was also true of conference abstracts which often reported on a high number of patients but without the necessary statistical calculations. Additionally, many papers failed to fulfill the inclusion criteria as they reported outcomes for patients with additional cancer types, for example cohorts of cancers of mixed gynecological subtypes or inclusion of gastrointestinal primary tumors.

Another significant limitation is the relatively small sample sizes analyzed in each paper. Ovarian cancer is not a common cancer, and in this review we accepted studies with a sample size of 5 or more patients. Although as we can see in Table 1 many studies included 50 or more patients by reviewing case notes over a period of 5 years or longer.

Given that MBO in advanced ovarian cancer patients often signals a terminal event, a key consideration must be quality of life. Although the primary outcome of this review and meta-analysis was median survival, aspects of quality of life were noted in the papers. For example, many authors commented on time taken to achieve symptom control or proportion of patients with symptoms controlled. Quality of life was variably defined, often by looking at only one particular symptom such as vomiting or pain, or by measuring what level of oral intake patients were able to tolerate. However, a comprehensive and standardized measure of health related quality of life (HRQOL) was not consistently used which would have been useful. This may prove difficult to achieve in future studies as we are concerned here with a frail group of patients who often are not physically or mentally equipped to complete long questionnaires.

A further problem is the issue of re-obstruction rates. Some studies have quoted up to 63% re-obstruction rates following surgery.^9^ For many patients with peritoneal seeding or bowel involvement, MBO can be a chronic state that waxes and wanes over time, perhaps as patients initially respond to management strategies including chemotherapy but then become resistant. Managing these patients is complex and will often utilize many different strategies, including surgical, interventional and supportive managements.

### What This Study Adds

This study provides a comprehensive review of the current knowledge base on MBO in advanced ovarian cancer. Robust statistical analysis give us a reliable indication of average survival and strong evidence upon which clinicians can draw when advising patients about their prognosis and facilitates decision making about clinical care with the patient. As no formal international guidelines exist for the management of this condition, this study can be drawn upon when devising these guidelines to standardize practice among different centers.

## Conclusion

Non-surgical management of malignant bowel obstruction in advanced ovarian cancer patients is an effective strategy to control symptoms, and survival outcomes are similar to those achieved by surgical management in this group of frail patients who are often at the end of life. There is currently insufficient evidence to facilitate the decision-making between different interventional strategies, and MDTs should therefore carefully tailor management to the individual patient’s clinical circumstances.
